# A review and comparison of breast tumor cell nuclei segmentation performances using deep convolutional neural networks

**DOI:** 10.1038/s41598-021-87496-1

**Published:** 2021-04-13

**Authors:** Andrew Lagree, Majidreza Mohebpour, Nicholas Meti, Khadijeh Saednia, Fang-I. Lu, Elzbieta Slodkowska, Sonal Gandhi, Eileen Rakovitch, Alex Shenfield, Ali Sadeghi-Naini, William T. Tran

**Affiliations:** 1 Department of Radiation Oncology, Sunnybrook Health Sciences Centre, Toronto, Canada; 2 Biological Sciences Platform, Sunnybrook Research Institute, Toronto, Canada; 3 Radiogenomics Laboratory, Sunnybrook Health Sciences Centre, Toronto, Canada; 4 Temerty Centre for AI Research and Education in Medicine, University of Toronto, Toronto, Canada; 5 Division of Medical Oncology, Department of Medicine, University of Toronto, Toronto, Canada; 6 Department of Laboratory Medicine and Molecular Diagnostics, Sunnybrook Health Sciences Centre, Toronto, Canada; 7 Department of Radiation Oncology, University of Toronto, Toronto, Canada; 8 Department of Engineering and Mathematics, Sheffield Hallam University, Sheffield, UK; 9 Physical Sciences Platform, Sunnybrook Research Institute, Toronto, Canada; 10 Department of Electrical Engineering and Computer Science, York University, Toronto, Canada; 11 Department of Radiation Oncology, University of Toronto and Sunnybrook Health Sciences Centre, 2075 Bayview Avenue, TB 095, Toronto, ON M4N 3M5 Canada

**Keywords:** Breast cancer, Electrical and electronic engineering

## Abstract

Breast cancer is currently the second most common cause of cancer-related death in women. Presently, the clinical benchmark in cancer diagnosis is tissue biopsy examination. However, the manual process of histopathological analysis is laborious, time-consuming, and limited by the quality of the specimen and the experience of the pathologist. This study's objective was to determine if deep convolutional neural networks can be trained, with transfer learning, on a set of histopathological images independent of breast tissue to segment tumor nuclei of the breast. Various deep convolutional neural networks were evaluated for the study, including U-Net, Mask R-CNN, and a novel network (GB U-Net). The networks were trained on a set of Hematoxylin and Eosin (H&E)-stained images of eight diverse types of tissues. GB U-Net demonstrated superior performance in segmenting sites of invasive diseases (AJI = 0.53, mAP = 0.39 & AJI = 0.54, mAP = 0.38), validated on two hold-out datasets exclusively containing breast tissue images of approximately 7,582 annotated cells. The results of the networks, trained on images independent of breast tissue, demonstrated that tumor nuclei of the breast could be accurately segmented.

## Introduction

Breast cancer remains the most commonly diagnosed type of cancer and the second most common cause of cancer-related death in women^1,2^. Improved patient survival requires a multidisciplinary approach, with specialists in pathology, medical, surgical, and radiation oncology^3^. Clinical management and accurate diagnosis of breast cancer type, staging, and grade require tissue biopsies. Biopsy specimens must be processed onto slides and stained using immunohistochemical (IHC) staining procedures^4^. Hematoxylin and Eosin (H&E) is a common stain^4^ used to identify nuclei and cytoplasm; Hematoxylin stains nuclei blue; Eosin stains the cytoplasm pink^4–6^. Examination of tissue biopsy specimens, prepared onto slides, is currently considered the gold-standard clinical diagnosis of cancer^6^. A recent update to the American Joint Committee on Cancer (AJCC) breast cancer staging manual^7^ now also incorporates histological features, such as grade and receptor status, into the prognostic staging process. However, the manual process of histopathological analysis is laborious, time-consuming, and limited by the quality of the specimen and the experience of the pathologist^8,9^. As a result, there has been growing interest in automating and digitizing pathological workflows, in addition to using computer-aided diagnosis (CAD) to streamline and optimize the process of tissue analysis^8^.

The emerging field of digital pathology has been made possible with the advent of high-resolution whole slide image (WSI) scanners^10^ and has further been driven by advancements in imaging technologies, data storage, and computer vision algorithms^11^. The high-resolution WSIs, obtained by scanning histopathological specimens, allow for in silico analysis. The application of artificial intelligence (AI) – in particular, its subfields of machine learning (ML) and deep learning (DL) – for in silico analysis are proving to be promising tools^10^. Researchers and data scientists in AI have applied convolutional neural networks (CNN) and machine classifiers to segment and classify objects, predict disease diagnosis, and treatment response; to tailor medical treatment, develop diagnostic assays, and determine patient response to treatment therapies^10^. For example, Wang et al.,^12^ demonstrated that in combining a CNN and random forest classifiers, mitotic nuclei could accurately be detected in H&E stained breast tissue. Digital pathology aims to develop clinically relevant computational methods to enhance specimen characterization and automate the detection of conventional features such as nuclear pleomorphism (e.g. nuclear shape and size) and tissue-spatial characteristics (e.g. cellular distribution)^13^.

Recent developments in CNN architectures represent the rapidly growing field of DL^14^. CNNs, trained on sizeable supervised image datasets, have been used to develop state-of-the-art learning algorithms^15^, capable of carrying out classification and segmentation tasks for medical image analysis. However, a significant challenge with advancing these algorithms includes the limited availability of supervised histopathological datasets. A dataset review is presented in Table 1 and outlines publicly available supervised histopathological datasets^16–25^. These datasets feature expert annotations of histopathological structure from a variety of tissues and staining techniques. A number of these datasets contain breast tissue samples; however, the method in which the histopathological structures were annotated is not consistent. Of the seven datasets that contain breast tissue, two datasets^17,21^ were not exhaustively annotated, and two other datasets^18,23^ contained annotations where the borders of the nuclei were not outlined. In general, training CNNs for semantic or instance segmentation requires exhaustive and full annotations of specific histopathological structures^19^.

**Table 1:** Open-source histopathological datasets.

Authors	Article	Tissue	Stain	Number of Images	Number of Annotated Nuclei	Type of Annotation	Exhaustive Annotations
Caicedo, J. et al.,	Nucleus segmentation across imaging experiments: the 2018 Data Science Bowl	A variety of organisms including: Human, Mice, and Fly	A variety of stains including: Fluorescent, Bright field, and H&E	841	37,333	Foreground	Yes
Drelie G. et al.,	A biosegmentation benchmark for evaluation of bioimage analysis methods	Breast	H&E	58	Unavailable	Foreground	No
Gamper, J. et al.,	PanNuke Dataset Extension, Insights and Baselines	Bladder, Ovarian, Pancreatic, Thyroid, Liver, Testis, Prostate, Stomach, Kidney, Adrenal gland, Skin, Head Neck, Cervix, Lung, Uterus, Esophagus, Bile-duct, Colon, Breast	H&E	7000 +	205,343	Foreground	Yes
Irshad, H. et al.,	Crowdsourcing image annotation for nucleus detection and segmentation in computational pathology: Evaluating experts, automated methods, and the crowd	Kidney, Renal	H&E	810	Unavailable	Foreground	Yes
Janowczyk, A. et al.,	Deep learning for digital pathology image analysis: A comprehensive tutorial with selected use cases	Breast	H&E	143	12,000	Foreground	No
Kumar et al.,	A Dataset and a Technique for Generalized Nuclear Segmentation	Breast, Liver, Kidney, Prostate, Bladder, Colon, Stomach, Lung, Brain	H&E	44	28,000	Foreground	Yes
Ljosa, V. et al.,	Annotated high-throughput microscopy image sets for validation	HT29 Colon-cancer	Fluorescent	12	Unavailable	Foreground	Yes
Martel et al.,	Assessment of Residual Breast Cancer Cellularity after Neoadjuvant Chemotherapy using Digital Pathology [Data set]	Breast	H&E	96	30,694	Point	Yes
Naylor, P. et al.,	Segmentation of Nuclei in Histopathology Images by Deep Regression of the Distance Map	Breast	H&E	50	4,022	Foreground	Yes
Wienert, S. et al.,	Detection and Segmentation of Cell Nuclei in Virtual Microscopy Images: A Minimum-Model Approach	Breast, Bone marrow, Liver, Kidney, Intestinal Mucosa	H&E	36	7,931	Point	Yes

The datasets were annotated in two ways, 1) foreground – borders of the nuclei were outlined, 2) point – nuclei were identified with a single mark. Exhaustive annotations indicate that all nuclei within the images had been marked or outlined.

U-Net^26^ is an example of a deep CNN (DCNN) that provides semantic segmentations. The network was first developed by Ronneberger et al., building on the fully convolutional network^27^ (FCN). U-Net improved on the FCN by reducing training time and enhancing localization with the addition of long skip connections and up-sampling layers to the encoder-decoder architecture. Since the introduction of U-Net, it is now recognized that the network can yield state-of-the-art segmentation results^28–31^.

Innovative segmentation algorithms that have gained widespread attention further include Mask regional convolutional neural network^32^ (Mask R-CNN), a two-stage DCNN. He et al.,^32^ introduced Mask R-CNN to provide instance segmentation, expanding on faster regional convolutional neural network^33^ (Faster R-CNN). At a high-level Mask R-CNN localizes each object instance to a bounding box then segments each instance.

Many recent state-of-the-art DCNN architectures, together with network weights pre-trained on large supervised image repositories, have been made publicly available for researchers and data scientists to implement. However, one of the restrictive factors in breast pathomics is the limited availability of supervised histopathological datasets. Previous studies^34,35^ and computer vision competitions^22,36^ have tested the generalizability of novel DCNN on multi-organ and multi-organism datasets; however, no study has evaluated if DCNNs trained on a variety of organs can accurately segment breast tumor nuclei. Therefore, this study aimed to determine if DCNNs, trained on histology images independent of breast tissue, can accurately segment tumor nuclei of the breast. Two DCNN architectures, one providing semantic segmentation (U-Net) and one providing instance segmentation (Mask R-CNN), will be evaluated. Additionally, classical segmentation methods will segment tumor nuclei of the breast, and the results compared to those of the DCNNs.

## Methods

### Training datasets

The training dataset implemented in this study featured open-source images from the Multi-Organ Nucleus Segmentation (MoNuSeg) challenge^16,37^. The MoNuSeg dataset, obtained from National Cancer Institute’s cancer genome atlas^38^ (TCGA), included high-resolution WSI of H&E stained slides from nine tissue types, digitized in eighteen different hospitals at 40 × magnification^16^. The types of tissue included breast, liver, kidney, prostate, bladder, colon, stomach, lung, and brain. Sub-regions (1,000 × 1,000 pixel), densely populated with nuclei, were extracted from the WSI. This study used the ground truth annotations released during the challenge. These ground truth annotations included all epithelial and stromal cells, which were annotated by students, then approved by an expert pathologist, with less than a 1% error in detection^16^. There were approximately 28,000 nuclei annotated across the entire dataset. The training set for this study consisted of colour normalized^39^ H&E images from all tissue types, excluding breast.

### Testing datasets

The hold-out (test) set exclusively contained H&E-stained images of breast tissue. There were fifty-eight images in total from two separate datasets. Eight images were from the MoNuSeg dataset and fifty from the Triple Negative Breast Cancer (TNBC) dataset^19^. The ground truth annotations associated with these images were curated and released with the respective dataset. The TNBC dataset contained fifty H&E stained images taken at 40 × magnification from eleven TNBC patients^19^. Three to eight sub-regions (512 × 512 pixel), with varying cellularity, were extracted per patient. The annotations were performed by three expert pathologists. Annotated cells included: normal epithelial cells, myoepithelial breast cells, invasive carcinoma cells, fibroblasts, endothelial cells, adipocytes, macrophages, and inflammatory cells. As the cell class was unavailable, with respect to both datasets, performance measures were based on segmenting all annotated cells within the test images.

All images were colour normalized^39^, then tiled to a size of 256 × 256 pixels with a 50% overlap. Post-processing the probability map, from any given U-Net like DCNN, involved calculating a threshold that maximized precision at a given intersections over union (IoU) threshold. Furthermore, we implemented morphological operations that filled missing pixels and removed small predicted artifacts.

### Software and hardware

All software related to this study was written in Python programming language version 3.7.6., using Anaconda (https://www.anaconda.com). Each DCNN was trained and implemented with Keras version 2.3.1^40^ and Tensorflow version 2.1.0^41^. U-Net like architectures were implemented with the segmentation models package (https://github.com/qubvel/segmentation_models) and Mask R-CNN was implemented with the matterport package (https://github.com/matterport/Mask_RCNN). The ImageJ2-Fiji^42,43^ (Fiji) package (https://imagej.nih.gov/ij/download.html) was applied for nuclei segmentation. Otsu threshold^44^ and watershed transform^45,46^ were applied with the scikit-image package (https://scikit-image.org) version 0.17.2. All experiments were performed on a workstation equipped with an AMD (Advanced Micro Devices, Inc., Santa Clara, USA) Ryzen Threadripper 1920X 12-Core Processor, 64 GB of RAM, and a single NVIDIA (NVIDIA Corporation, Santa Clara, USA) GeForce RTX 2080 Ti graphics processing unit (GPU).

### Classical segmentation techniques

Fiji is an open-source image processing and analysis software package that features a built-in nuclei segmentation pipeline. To prepare the images for segmentation, they were first colour normalized^39^, then converted to gray-scale. The pipeline involved implementing a Gaussian filter, thresholding the images, followed by applying the watershed transform. The classical segmentation techniques (Otsu threshold, Watershed transform, Fiji) applied to the images of this study, were used to compare the performance of DCNNs to such techniques.

### U-net

Eight DCNNs with U-Net like architecture were trained for this study; furthermore, the encoders of the DCNNs were replaced with the following networks: visual geometry group^47^ (VGG)-16, VGG-19^47^, Residual Networks^48^ (ResNet)-50, ResNet-101^48^, ResNet-152^48^, Dense convolutional Network^49^ (DenseNet)-121, DenseNet-201^49^, and Inception-v3^50^. These DCNN encoders were chosen because of their success in image classification^14,51^ and their wide use in a range of instance and semantic segmentation tasks^52–54^. Weights pre-trained on the ImageNet^55^ dataset initialized each DCNN. All DCNN decoders consisted of up-sampling, concatenation of the respective feature map from the encoding path, followed by two blocks of 3 × 3 convolutions, batch normalization^56^, and rectified linear units^57^ (ReLU) activation. The final layer of all DCNNs consisted of a 1 × 1 convolution with SoftMax activation.

All U-Net like DCNNs implemented training images with randomly applied combinations of rotation, distortion, skew, shear, and flip augmentations. The normalized training images was passed through an augmentation pipeline in groupings of H&E images, annotations, and weighted maps; ensuring that all corresponding images remained spatially aligned. All images were further tiled to a size of 256 × 256 pixels, with a 10% overlap.

There were 6,720 images used during training, split into a ratio of 80:20 between the training and validation sets, respectively. Adaptive moment estimation^58^ (Adam) optimizer with a learning rate of 1e-4 was used to fine-tune all U-Net like DCNNs. All DCNNs were trained until convergence. Each DCNN had a combined loss function of weighted cross-entropy^26^ and soft Dice (Eq. 1):

1 $${\mathcal{L}}_{combined}= {\mathcal{L}}_{WCE}+ {(1-\mathcal{L}}_{Dice})$$

Equation (2), which represents weighted cross-entropy^26^ was formulated as:

2 $${\mathcal{L}}_{WCE}= -\sum_{\mathcal{X}\in {\mathbb{X}}}{\mathcal{W}}_{map}\left(x\right) \mathrm{log}({p}_{a(x)}(\mathcal{X}))$$

where $\mathcal{X}$ was the image used during training, $\alpha$ was the ground truth annotation of each image, across all classes, and $\mathcal{W}$ was the weighted map introduced to the loss function. Dice loss (Eq. 3) was formulated as:

3 $${\mathcal{L}}_{Dice}= \frac{2\sum_{\mathcal{X}\in {\mathbb{X}}}p\left(\mathcal{X}\right)gt\left(\mathcal{X}\right)}{\sum_{\mathcal{X}\in {\mathbb{X}}}({p(x)}^{2}+ {gt\left(\mathcal{X}\right)}^{2})}$$

where $\mathrm{\rm P}$ was the output of the SoftMax activation function, and $gt$ was the ground truth annotation.

### Mask R-CNN

Prior to training Mask R-CNN, all training images were tiled to a resolution of 256 × 256 pixels with a 10% overlap, for a total of 560 images. The images were further randomly split into training and validation sets at a ratio of 80:20, respectively. The CNN backbone of Mask R-CNN was ResNet-101 and through optimization, Gradient Descent^59^ was chosen as the optimizer for the network. Furthermore, ResNet-101 was initialized with weights pre-trained on the common objects in context^60^ (COCO) dataset. Gradient Descent was set to a momentum to 0.9, a learning rate of 1e-3, a weight regularizer of 1e-4, and trained until convergence.

### Ensemble networks

U-Net ensemble calculated the pixel-wise mean of the probability maps from the U-Net like DCNNs with VGG-19, ResNet-101, and DenseNet-121 encoders.

**Figure 1:**
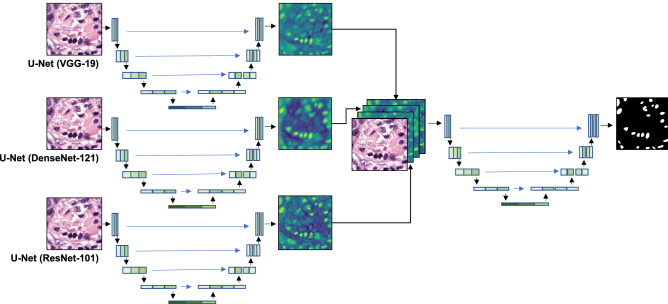
Network architecture of GB U-Net, a gradient boosting network. The first stage of GB U-Net concatenated features maps from three U-Net like DCNN (VGG-19, DenseNet-121, and ResNet-101 encoders), with the colour normalized H&E image. The second stage of GB U-Net passed the concatenated image through a final U-Net. The original H&E images were curated by the TCGA. Ground truth annotations were released by Kumar et al.^16^, and additional annotations were released during the MoNuSeg challenge.

A gradient boosting network (GB U-Net) was developed to improve localization. GB U-Net is a two-stage network where at a high-level, concatenates feature maps from U-Net like DCNNs with VGG-19, ResNet-101, and DenseNet-121 encoders with the colour normalized H&E image, then uses the concatenated images as input to an additional U-Net (Fig. 1). The feature maps concatenated with the H&E image were taken from the last convolutional layer, just before the SoftMax activation. The second stage of GB U-Net was trained with a slightly modified encoder, which featured repeated blocks of 3 × 3 convolutions, batch normalization^56^, ReLU^57^ activation, and Dropout^61^ layers. The decoder followed the same structure as all previous U-Net like DCNNs. GB U-Net was trained until convergence using the 6,720 augmented H&E images, masks, and weighted maps previously mentioned. The network featured a combined loss function of weighted cross-entropy^26^ and soft Dice, previously outlined.

### Evaluation metrics

This study's evaluation metrics were Aggregated Jaccard Index^16^ (AJI) and mean average precision (mAP). Kumar et al.,^16^ proposed AJI, which penalized pixel and object level detection. AJI penalized missed objects, falsely detected objects, along with under and over segmented objects. It did this by computing the ratio of aggregated cardinality of intersection and union of predicted nuclei matched with annotated nuclei. The mAP is a popular metric used in object detection and segmentation tasks. The metric yields average precision over multiple IoU thresholds. Specifically, ten IoU thresholds starting at 0.5 and increasing linearly by 0.05 to 0.95, were used for this study.

## Results

Table 2a,b summarize the results of the DL and classical segmentation methods for the MoNuSeg and TNBC datasets, respectively. Table 2a is composed of three categories, 1) classical segmentation methods (Otsu threshold, watershed transform, Fiji), 2) U-Net like DCNNs with varying encoders, 3) Mask R-CNN and ensemble networks. With an AJI of 0.3396 and mAP of 0.237, Fiji considerably outperformed the Otsu thresholding and watershed transform. However, the DL methods outperformed Fiji across all metrics. In comparing all U-Net like DCNNs, the Densenet-201 encoder scored highest in AJI with 0.5083, while the ResNet-101 encoder scored highest in mAP with 0.3318. With the highest AJI, the Densenet encoder surpassed all U-Net like DCNNs in both object level and pixel-wise segmentation accuracy. In contrast, the ResNet encoder demonstrated that it excelled in accurately classifying nuclei from other histopathological structures. Furthermore, the DenseNet-201 encoder scored highest in F1 at an IoU threshold of 0.5 and recall at IoU thresholds of 0.5 and 0.7; demonstrating that the network excelled in correctly identifying all nuclei, while also provided the best balance in correctly identifying and separating nuclei from other structures. With regards to the TNBC dataset (Table 2b) the ResNet-101 encoder scored highest in AJI with 0.5080, mAP with 0.3306, and AP at both IoU thresholds.

**Table 2:** F1, recall, and precision metrics are reported for two intersection over union thresholds, 0.5 and 0.7.

Segmentation Methods	Aggregated Jaccard Index	Mean Avg. Precision	F1(0.7)	Recall(0.7)	Precision(0.7)	F1(0.5)	Recall(0.5)	Precision(0.5)
**(a) Nuclei segmentation results for the MoNuSeg test dataset**								
Otsu	0.0456	0.0677	0.0310	0.0255	0.0396	0.1619	0.1331	0.2065
Watershed	0.0828	0.1581	0.0863	0.0591	0.1594	0.2743	0.1880	0.5070
Fiji	**0.3396**	**0.2370**	**0.1828**	**0.1447**	**0.2481**	**0.4411**	**0.3493**	**0.5986**
U-Net(VGG-16)	0.4925	0.2736	0.2886	0.2927	0.2845	0.6511	0.6604	0.6420
U-Net(VGG-19)	0.4841	0.3007	0.3452	0.3480	0.3426	0.6735	0.6788	0.6683
U-Net(ResNet-50)	0.4882	0.3163	**0.3772**	0.3884	0.3667	0.6967	0.7173	0.6772
U-Net(ResNet-101)	0.4687	**0.3318**	0.3242	0.2834	**0.3788**	0.6133	0.5360	**0.7166**
U-Net(ResNet-152)	0.4396	0.3119	0.3368	0.3241	0.3506	0.6706	0.6452	0.6980
U-Net(DenseNet-121)	0.4668	0.2988	0.3579	0.3738	0.3432	0.6796	0.7099	0.6517
U-Net(DenseNet-201)	**0.5083**	0.3185	0.3760	**0.3884**	0.3645	**0.6980**	**0.7208**	0.6765
U-Net(Inception-v3)	0.4440	0.2879	0.3044	0.3005	0.3085	0.6422	0.6339	0.6507
Mask R-CNN	0.5282	0.3884	**0.4028**	0.3518	**0.4773**	0.6648	0.5813	0.7859
U-Net Ensemble	0.4926	0.3381	0.3791	**0.3677**	0.3913	**0.6957**	**0.6746**	0.7180
GB U-Net	**0.5331**	**0.3909**	0.4007	0.3509	0.4669	0.6862	0.6010	**0.7997**
**(b) Nuclei segmentation results for the TNBC dataset**								
U-Net(VGG-16)	0.3538	0.1672	0.1614	0.1581	0.1648	0.5042	0.4940	0.5149
U-Net(VGG-19)	0.3829	0.1742	0.1364	0.1226	0.1536	0.5099	0.4585	0.5741
U-Net(ResNet-50)	0.3972	0.2324	0.2701	0.2530	0.2897	0.5638	0.5281	0.6048
U-Net(ResNet-101)	**0.5080**	**0.3306**	0.2904	0.2214	**0.4217**	0.5427	0.4139	**0.7882**
U-Net(ResNet-152)	0.4063	0.2594	0.2790	0.2478	0.3194	0.5874	0.5216	0.6723
U-Net(DenseNet-121)	0.4246	0.2929	0.3600	**0.3327**	0.3922	0.6216	0.5745	0.6772
U-Net(DenseNet-201)	0.4453	0.2843	**0.3604**	0.3287	0.3988	**0.6298**	**0.5745**	0.6970
U-Net(Inception-v3)	0.3817	0.1962	0.1562	0.1303	0.1947	0.4703	0.3925	0.5864
Mask R-CNN	0.4899	0.3449	**0.4392**	**0.4027**	0.4830	**0.6732**	**0.6172**	0.7403
U-Net Ensemble	0.4836	0.2808	0.2849	0.2430	0.3442	0.6068	0.5176	0.7331
GB U-Net	**0.5403**	**0.3772**	0.4205	0.3540	**0.5176**	0.6581	0.5541	**0.8102**

Bolded values identify the highest scoring segmntation method within 1)classical segmentation, 2)U-Net like DCNNs, 3)Mask R-CNN and ensemble networks, with respect to the evaluation metric.

The overall top-scoring network in both datasets was GB U-Net. For the MoNuSeg dataset GB U-Net achieved an AJI of 0.5331 and a mAP of 0.3909, while for the. TNBC dataset the network achieved an AJI of 0.5403 and mAP of 0.3772.

**Figure 2:**
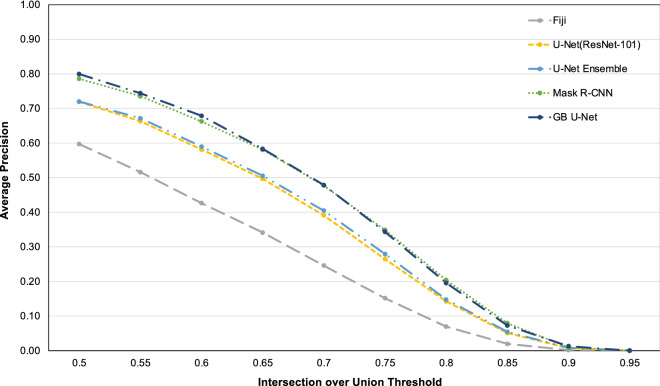
Average precision graphically displayed, across ten intersection over union thresholds, for the MoNuSeg test dataset.

Figure 2 displays AP plotted against the evaluated IoU thresholds for several segmentation methods using the MoNuSeg test dataset. GB U-Net and Mask R-CNN scored highest in this metric. Both networks displayed similar AP values throughout the IoU thresholds; however, GB U-Net displayed superior performance in the lower thresholds, while Mask R-CNN performed better in thresholds greater than 0.65. Both the ResNet-101 encoder and U-Net Ensemble displayed similar mAP scores, and Fig. 2 also demonstrated that both AP values remained similar across all IoU thresholds. A noticeable difference between the networks was that U-Net ensemble scored slightly higher in AP throughout the mid-range of IoU thresholds (0.55–0.8). Additionally, Fig. 2 demonstrated that the DCNNs outperformed the top-scoring classical segmentation method (Fiji) across all IoU thresholds.

**Figure 3:**
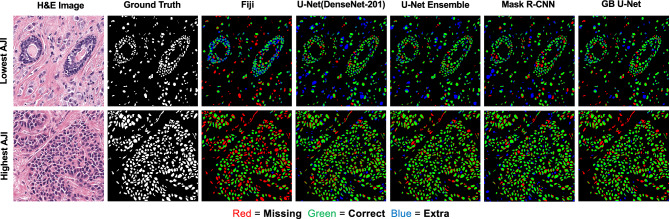
Nuclei annotations of the lowest and highest AJI images of the MoNuSeg dataset. Annotations pertaining to classical segmentation methods and the DCNN have been colour coded, such that green indicated true positive, red indicated false negative, and blue indicated false positive pixels. The original H&E images were curated by the TCGA. Ground truth annotations were released by Kumar et al.^16^, and additional annotations were released during the MoNuSeg challenge.

Figure 3 displays the predicted maps of the MoNuSeg test images with the lowest and highest combined AJI. The predicted maps were colour coded with green indicating correctly classified pixels (true positive), red indicating pixels not classified as nuclei (false negative), and blue indicating pixels that were miss classified as nuclei (false positive). Row 1 (Fig. 3) provided examples of predicted maps associated with the lowest scoring image. The AJI values of these images are summarized in Table 3. The image was difficult to segment accurately, as large areas of the image displayed densely crowded regions of tumor nuclei. Fiji struggled to correctly separate nuclei within these areas, which resulted in over-segmentation. However, Fiji also under-segmented the surrounding areas. The DCNNs were better able to separate nuclei within the crowded areas; however, the networks tended to over-segment the outer areas. The top-scoring networks, GB U-Net and Mask R-CNN performed similarly on both images; however, under qualitative analysis, the main difference between the networks was that Mask R-CNN tended to over-segment images compared to GB U-Net.

**Table 3:** Individual Aggregated Jaccard Index scores of the eight breast tissue images, which composed the MoNuSeg test dataset.

Image ID	Fiji	U-Net (DenseNet-201)	Ensemble U-Net	Mask R-CNN	GB U-Net
TCGA-A7-A13E-01Z-00-DX1	0.35	0.515	0.579	0.517	0.628
TCGA-A7-A13F-01Z-00-DX1	0.328	0.434	0.491	0.554	0.518
TCGA-AC-A2FO-01A-01-TS1	0.252	0.493	0.478	0.543	0.511
TCGA-AO-A0J2-01A-01-BSA	0.323	0.561	0.486	0.56	0.535
TCGA-AR-A1AK-01Z-00-DX1	0.31	0.526	0.458	0.511	0.5
TCGA-AR-A1AS-01Z-00-DX1	0.634	0.623	0.632	0.611	0.676
TCGA-E2-A14V-01Z-00-DX1	0.272	0.482	0.436	0.5	0.464
TCGA-E2-A1B5-01Z-00-DX1	0.249	0.433	0.379	0.43	0.432

## Discussion

This was the first study that investigated DCNN’s accuracy in segmenting invasive carcinoma of the breast when trained on H&E-stained digital images of liver, kidney, prostate, bladder, colon, stomach, lung, and brain cancers. The eleven DCNNs trained for this study included: eight U-Net like architectures with various encoders, two gradient boosting networks, and Mask R-CNN. Furthermore, the study's three classical segmentation methods included: Otsu threshold, watershed transform, and Fiji. Overall, the DCNNs outperformed all classical segmentation methods. The top-scoring DCNN in AJI and mAP was GB U-Net. Mask R-CNN scored slightly lower; however, it outperformed all U-Net like DCNNs in the MoNuSeg test dataset. Segmentation performance of the DCNNs demonstrated that, in using transfer learning, the networks were able to effectively implement features learned independently of breast tissue to segment tumor nuclei within breast tissue.

The work presented in this study reflects the emerging field of pathomics, which combines digital pathology and novel AI-based software. Overall, pathomics-based approaches provide novel techniques to augment clinical workflows by automating WSI feature extraction for diagnostic, treatment, and prognostic applications. For example, breakthroughs in AI-based technologies have automated histological grading of breast cancer specimens^62^, mitosis detection^12^, nuclei pleomorphism segmentation^63^, and tubule nuclei classification of estrogen receptor (ER) + breast cancers^64^. Furthermore, DL networks have automated important diagnostic and prognostic factors such as the classification of human epidermal growth factor receptor 2 (HER2) score^65^, along with ER and progesterone receptor (PR) status^66^. These molecular markers are fundamental to clinical decision making both at the time of diagnosis and recurrence, if recurrence occurs, to select the most effective therapies. Recent work has also identified certain tumor nuclei morphological features to be independent prognostic factors for tumor size and tubular formation^67^, alongside features that are significantly associated with eight-year disease-free survival^68^. Additionally, recent work by Ali et al.,^69,70^ introduced a prognostic assay that evaluated lymphocyte density as a predictor of pathological complete response (pCR) in breast cancer patients with neoadjuvant chemotherapy regimens. As the field of oncology moves into an era of precision medicine^71^, novel insights and innovations in tumor nuclei segmentation and feature extraction may lead to more precise breast cancer treatment selection. One such way is AI driven diagnostic and theragnostic tools that leverage DL architectures.

U-Net excels in biomedical semantic segmentation^29^; however, the network struggles with correctly classifying close or touching objects. In semantic segmentation, each pixel is identified with a class label specific to the object. Object instances within the same class are not separated; therefore, all foreground objects will possess the same class label within binary segmentation. Objects that are close together or overlapping, i.e., areas of crowded nuclei, may be incorrectly classified as a single object. Weighted cross-entropy^26^ is a loss function used to improve pixel classification of close or touching objects. Pre-calculated weighted maps^26^, are passed to the network and used to weight the cross-entropy loss. Weighted cross-entropy severely penalizes incorrect pixel classification of close or touching nuclei.

Symmetrical networks such as U-Net’s encoder-decoder architecture further possess an opportunity to modify the network’s structure and improve performance. The original U-Net encoder followed a typical CNN structure, where successive convolution, activation, and pooling layers calculated feature maps. However, the complexity of training DCNNs has resulted in the advancement of network architectures^15^ and contributed to the development of novel networks such as: VGG^47^, ResNet^48^, DenseNet^49^, and GoogLeNet^50^. These relatively newer networks have achieved superior state-of-the-art object detection results^14^ with improvements in feature extraction, minimizing the vanishing gradient, and aiding network convergence. By modifying the network architecture or replacing the standard encoder with a deeper CNN and using U-Nets long skip connections, researchers have improved object and segmentation level performance^52,53,72^.

Mask R-CNN is a novel network that was proposed to provide instance level annotations. The first stage of Mask R-CNN is identical to region proposal networks^33^ (RPN) of Faster-RCNN, which proposes class probability maps. The CNN backbone of Mask R-CNN provides the feature map as input for the convolutional layer specific to RPN; the convolutional layer's output then feeds both regression and classification layers. The regression layer predicts region proposals, while the classification layer predicts the probability of an object bound within the proposal. The second stage of Mask R-CNN uses the region proposals to calculate the object class, bounding box offset, and segments the object using the FCN^27^. Region of interest (RoI) align was introduced with Mask R-CNN to correct the RoI pool's roundoff error, aligning the segmented image with the object. RoI pool, introduced with fast regional convolutional neural network^73^ (Fast R-CNN), was developed to reduce computational time by taking feature maps, defined by a RoI, and scaling them to a fixed size; this prompted misalignment between the feature map and input image. RoI align incorporated bilinear interpolation to calculate floating-point values of the sampling points, avoiding quantization. Mask R-CNN outputs bounding box coordinates, object class probability score, and a segmented map of each object instance.

ML-based approaches work exceptionally well when the training and testing data are drawn from the same feature space^74^. In circumstances where data are sparse, transfer learning can transfer knowledge from one domain to a task within an independent domain^74^. However, it is imperative to ensure that both domains are related to avoid negative transfer. Transfer learning has been extensively implemented by data scientists for tasks such as text classifiers^75–77^. However, this was the first study investigating the use of transfer learning to segment tumor nuclei of breast tissue exclusively, to the author's knowledge. Currently there are minimal open-source annotated breast tissue datasets (Table 1), therefore it is essential to identify if DCNNs can leverage transfer learning to accurately segment breast nuclei from open-source datasets. Previous studies^34,35,78^ have used the MoNuSeg and TNBC datasets to evaluate the segmentation accuracy of novel DCNNs. The MoNuSeg test dataset of these studies included fourteen tissue samples from seven organs, three (bladder, colon, stomach) of which were withheld from the training set. Specifically, work by Graham et al^78^., compared a novel DCNN (HoVer-Net) to various other DCNNs. In the first experiment Graham and colleagues trained the DCNNs using the MoNuSeg dataset. In comparing the results of the current study to those of Graham and colleagues, GB U-Net outperformed Cell Profiler^79^ (AJI: 0.366), QuPath^80^ (AJI: 0.432), FCN8^27^ (AJI: 0.281), FCN8 + WS (AJI: 0.429), SegNet^81^ (AJI: 0.377), SegNet + WS (AJI: 0.508), deep contour-aware network (DCAN)^82^ (AJI: 0.525), and CNN3^16^ (AJI: 0.508). In the second experiment Graham and colleagues evaluated the DCNNs using the TNBC dataset. In comparing their results to the current study GB U-Net outperformed FCN8 + WS (AJI: 0.506), FCN8 (AJI: 0.281), Mask R-CNN (AJI: 0.529), DCAN (AJI: 0.537), Micro-Net^83^ (AJI: 0.531), and DIST^19^ (AJI: 0.523). Furthermore, a study by Wang et al^34^., evaluated various DCNNs trained using the MoNuSeg dataset. In comparing their results to the current study GB U-Net, again, outperformed FCN (AJI: 0.452), U-Net^26^ (AJI: 0.513), SegNet (AJI: 0.505), and DCAN (AJI: 0.518). Additionally, a study by Liu et al^35^., used thirteen images from the TNBC dataset to evaluate multiple DCNNs. In comparing their results to the current study GB U-Net outperformed GCN^84^ (AJI: 0.1907), Mask R-CNN (AJI: 0.5297), and pixel2pixel^85^ (AJI: 0.4760). However, novel DCNNs including HoVer-Net (AJI: 0.618 & 0.59), Bending Loss^34^ (AJI: 0.641), and panoptic segmentation^35^ (AJI: 0.5865) achieved a higher AJI compared to GB U-Net.

Many of the studies mentioned above featured DCNNs developed to provide precise segmentation of tumor nuclei, explicitly improving on segmenting areas of densely populated nuclei. Differentiation of nuclei is essential for spatial features; however, a study by Boucheron et al^86^., identified that, specific to tumor nuclei classification, perfectly segmented nuclei do not guarantee optimum classification accuracy. Although GB U-Net did not outperform all DCNNs the results demonstrate that transfer learning can be implemented while training DCNNs to segment breast tumor nuclei if precise segmentations are not explicitly required.

A limitation of this study is the relatively small dataset used for training and testing, based on the limited open-source datasets. Future work will involve expanding the number of images and types of tissues included in the dataset. Generative adversarial networks (GANs) are an innovative approach to creating synthetic histopathological images. GANs are a promising approach to data synthesis, in which synthetic H&E stained histopathology images and ground truth images can be generated from features learned from the training data^87–89^. GANs have the potential to significantly increase histopathological training sets without requiring additional annotations from expert pathologists.

## Conclusion

The study's objective was to determine if transfer learning could be implemented with DCNNs to segment tumor nuclei of breast tissue accurately. This study introduced a novel ensemble network (GB U-Net), which scored highest in AJI and mAP. Mask R-CNN scored slightly lower in AJI and mAP, compared to GB U-Net; however, one of the main differences between the networks was that Mask R-CNN displayed improved AP at higher IoU thresholds (greater than 0.65). Overall, this study demonstrated that DCNNs trained on images independent of breast tissue could accurately segment invasive carcinoma of the breast by implementing transfer learning. One of the main limitations facing DL based researchers and data scientists is the limited availability of training and testing data. As it is impractical to expect that expert pathologists provide the time commitment required to produce large-scale histopathological datasets, various boosting and data synthesis options, such as transfer learning and GANs, should be further explored to augment clinical workflows and ultimately lead to improved patient care.

## Data Availability

The MoNuSeg dataset used for the current study is available in the Medical Image Computing and Computer Assisted Intervention Society (MICCAI) 2018, MoNuSeg challenge repository, https://monuseg.grand-challenge.org/Data/. The TNBC dataset used for the current study is available in the segmentation of nuclei in histopathology images by deep regression of the distance map, Peter Jack Naylor GitHub repository, https://github.com/PeterJackNaylor/DRFNS. The authors have made every effort to provide a detailed description of the software and hardware implemented with this study. All data, which has not been published in tables or alongside the article will be made available from the corresponding author upon request.
